# Aptamer-Based In Vivo Therapeutic Targeting of Glioblastoma

**DOI:** 10.3390/molecules25184267

**Published:** 2020-09-17

**Authors:** Valeriana Cesarini, Chiara Scopa, Domenico Alessandro Silvestris, Andrea Scafidi, Valerio Petrera, Giada Del Baldo, Angela Gallo

**Affiliations:** 1 RNA Editing Laboratory, Oncohaematology Department, IRCCS Ospedale Pediatrico Bambino Gesù (OPBG), 00146 Rome, Italy; valeriana.cesarini@opbg.net (V.C.); chiara.scopa@opbg.net (C.S.); dalessandro.silvestris@opbg.net (D.A.S.); andrea.scafidi@opbg.net (A.S.); valerio.petrera@opbg.net (V.P.); giada.delbaldo@opbg.net (G.D.B.); 2 Institute of Translation Pharmacology, National Research Council of Italy (CNR), 00133 Rome, Italy

**Keywords:** GBM, GBM therapy, aptamers, nanoparticles, drug delivery

## Abstract

Glioblastoma (GBM) is the most aggressive, infiltrative, and lethal brain tumor in humans. Despite the extensive advancement in the knowledge about tumor progression and treatment over the last few years, the prognosis of GBM is still very poor due to the difficulty of targeting drugs or anticancer molecules to GBM cells. The major challenge in improving GBM treatment implicates the development of a targeted drug delivery system, capable of crossing the blood–brain barrier (BBB) and specifically targeting GBM cells. Aptamers possess many characteristics that make them ideal novel therapeutic agents for the treatment of GBM. They are short single-stranded nucleic acids (RNA or ssDNA) able to bind to a molecular target with high affinity and specificity. Several GBM-targeting aptamers have been developed for imaging, tumor cell isolation from biopsies, and drug/anticancer molecule delivery to the tumor cells. Due to their properties (low immunogenicity, long stability, and toxicity), a large number of aptamers have been selected against GBM biomarkers and tested in GBM cell lines, while only a few of them have also been tested in in vivo models of GBM. Herein, we specifically focus on aptamers tested in GBM in vivo models that can be considered as new diagnostic and/or therapeutic tools for GBM patients’ treatment.

## 1. Introduction

GBM or astrocytoma grade IV is the most aggressive, infiltrative, and lethal brain tumor in humans [1]. GBM is currently incurable, due to its resistance to conventional therapies and invasive nature. Despite advances in therapeutic options, the prognosis remains very poor due to the lack of safe and effective carriers able to specifically target tumor cells and to penetrate into the tumor [2].

In the past decade, much attention has been focused on aptamers that are emerging as safe delivery vehicles for targeted cancer therapeutics. Aptamers are short single-stranded DNA or RNA oligonucleotides able to bind targeted molecules with high affinity in a three-dimensional shape [3]. Aptamers against a specific target can be generated via an in vitro selection process called Systematic Evolution of Ligands by EXponential enrichment (SELEX). The conventional SELEX method mainly consists of three steps: selection, partitioning, and amplification. Before the selection step, a library of oligonucleotides (DNA or RNA) is synthesized, with generally up to 1015 different unique sequences [4]. Each unique sequence contains random bases (20–50 nt) flanked by two conserved primer-binding sites, which are used for the PCR amplification step. During selection, the library is incubated with purified target molecules for a specific time; then, the unbound sequences are eliminated (partitioning step), while the target-bound sequences are isolated and directly amplified by PCR (DNA SELEX) or reverse transcribed to amplifiable cDNA and subsequently transcribed back to RNA using T7 RNA polymerase (RNA SELEX) [4,5]. The PCR products are utilized for the next round of selection. After several selection rounds, the enriched sequences are sequenced, and their binding abilities are further evaluated. After SELEX, the aptamer sequences can be modified to improve their binding affinity and selectivity, thus reducing off-target effects [6].

**Figure 1:**
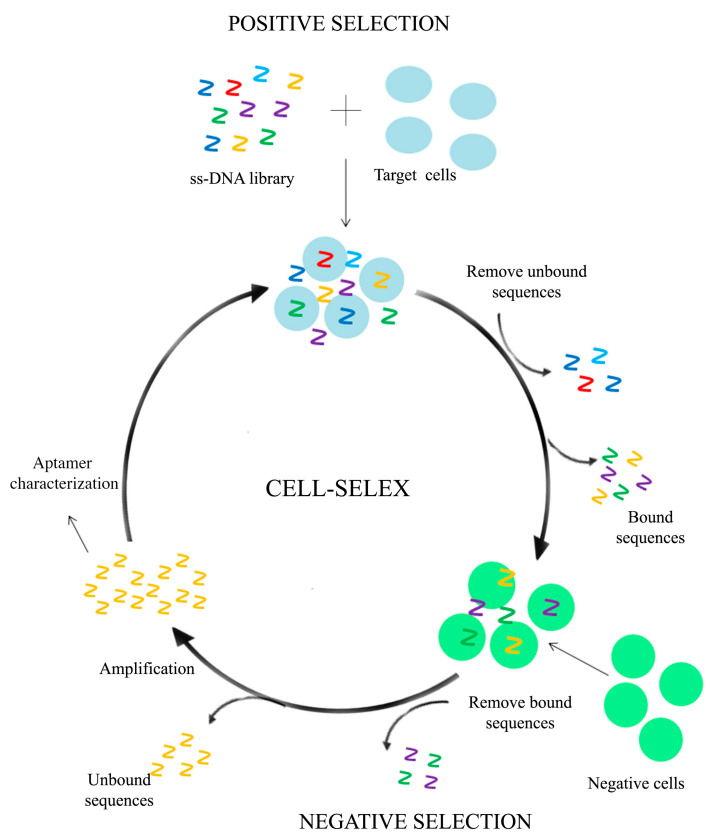
Schematic representation of a Cell-Systematic Evolution of Ligands by SELEX method. Initially, a library of oligonucleotides is incubated with the target cells. The unbound sequences are removed by washing, while the bound sequences are collected. After an incubation with the negative cells, the bound sequences are discarded, while the unbound sequences are collected and amplified by PCR. The PCR products are utilized for the next round of selection. After several selection rounds, the enriched sequences are sequenced and characterized [47].

The cell-SELEX is a modification of the traditional SELEX process using whole living cells as target instead of isolated target molecules. Specifically, a library of oligonucleotides is incubated with the target cells and the unbound sequences are removed by washing, while the bound sequences are collected. These sequences are able to bind specific molecules on the cell surface (positive selection). After incubation with the “negative/non-cancerous” cells, a further selection of specific aptamers is performed (negative selection) and the selected aptamer utilized for the next round of selection (Figure 1).

The in vitro classical SELEX method, with purified proteins, has the clear advantage to obtain optimal enrichment during the selection process; the cell-SELEX method is preferred, for example, when the target is “unknown” or cell type-specific. Of note, in some cases the target protein may be partially hidden and/or unreachable in vivo, thus the whole living cells represent a more physiological condition. Since most cancer cells express high specific surface markers used for diagnosis and therapy, cell-SELEX technology plays a significant role in cancer biology. Thanks to this approach, the development of aptamer against specific cancer cell receptors/proteins is highly specific, creating the opportunity for targeted and personalized treatments [7,8].

Beside the high specificity to the target, the aptamers possess other key important characteristics that make them unique as therapeutic molecules, namely the low immunogenicity and their small size that further increase their ability of tissue penetration [9].

To date, a multitude of aptamers have been generated against cell membrane proteins expressed on GBM cells generated by both in vitro and cell-based SELEX methods [10]. Several GBM cell lines were used for aptamer selection. Some of the aptamers were isolated via selection against known specific GBM markers, whereas others were identified by selection against target cell surface molecules (cell-SELEX) [11]. These aptamers were used to specifically deliver diagnostic or therapeutic agents for GBM diagnosis and treatment [6]. Moreover, “imaging agents” can also be conjugated to aptamers to allow the development of more effective tumor imaging strategies for both histological analysis of tissues and in situ tumor location helping surgical resection [12,13,14,15].

Specifically, imaging techniques are used in all phases of cancer management, providing complementary information to patho-physiological diagnosis, allowing for improved cancer staging and therapy planning. In brain tumors, magnetic resonance imaging (MRI) and positron emission tomography (PET), as a complementary technology, represent the standard methods for defining lesion boundaries [16] and for a more precise identification of GBM borders from the surrounding normal brain tissue. Aptamers, coupled with some imaging agents such as fluorescent or radionuclide labels, bioconjugates, and nanoparticles (NPs), have been developed to improve surgical removal, representing an advanced imaging tool for the in vivo tumor visualization [17,18,19,20,21,22].

Additionally, the GBM-targeted aptamers have also been employed for the isolation and enrichment of GBM cells from biological samples [23,24,25].

Aptamers have been largely tested in vitro, showing multiple anticancer properties in GBM cell lines [10,26,27,28,29,30,31,32,33,34,35]. However, only some of the GBM aptamers, able to reach the tumor site in vivo, are capable of efficiently treating this cancer [36,37,38,39,40,41,42,43,44,45,46].

**Figure 2:**
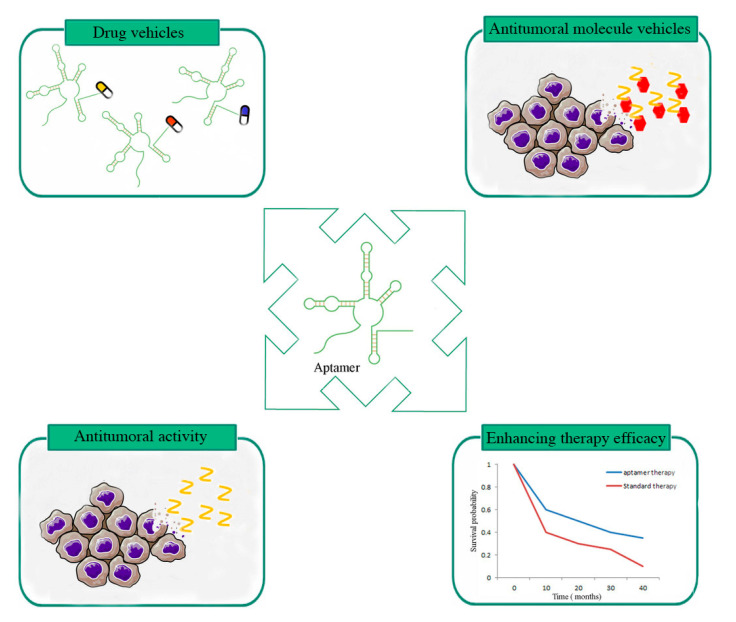
Schematic representation of aptamers in in vivo applications.

Herein, we summarize the studies involving specific GBM aptamers whose therapeutic efficacy was tested and confirmed in GBM in vivo models (Figure 2 and Table 1).

## 2. Aptamers Showing In Vivo Therapeutic Effects

A large number of aptamers have been generated against GBM cell membrane proteins [47]. Some of these aptamers were developed as anti-GBM tools and/or tested as a vehicle for antitumoral molecules blocking the activity of GBM receptors involved in gliomagenesis and cancer progression (Table 1).

### 2.1. Aptamers Used as Antitumoral Molecules

#### 2.1.1. Gint4.T and CL4 Aptamers

The platelet-derived growth factor receptor beta (PDGFRβ) is overexpressed in GBM cells and is involved in cell migration [42]. Camorani and co-authors [42], by using U87MG GBM cells, selected a PDGFRβ-specific RNA aptamer (Gint4.T) after 14 differential whole cell-SELEX rounds. Gint4.T has been tested in U87MG cells, showing that it is able to reduce migration and proliferation. Specifically, Gint4.T induced an S-phase cell-cycle arrest and stimulated the differentiation of the U87MG cells [42].

The anti-EGFR pro-apoptotic CL4 is an aptamer raised against the epidermal growth factor receptor (EGFR) and found to have a strong cytotoxic effect in EGFR positive cancer cells [48]. CL4 was also tested in vivo in U87MG-derived mouse xenografts. The aptamer was administered intravenously at days 0, 3, 5, and 7, leading to a significant reduction in tumor growth [42].

Interestingly, the anti-GBM properties of Gint4.T/CL4 combined aptamers were compared with three commercial anticancer drugs—gefitinib and cetuximab (both drugs against EGFR) and imatinib (against PDGFR) [49]. Dose- and time-dependent experiments showed that T98G and U87MG cells treated with gefitinib and cetuximab are extremely resistant to both the anticancer therapeutic agents. Differently, Gint4.T + CL4 treatment reduced (70%) cell viability similarly to the temozolomide treatment, which is, at present, the conventional GBM therapy [50,51].

In order to assess the in vivo targeting of Gint4.T, mice bearing xenografts from luciferase-expressing U87MG cells were treated with the aptamer single intravenous injection. Bioluminescence assay demonstrated that Gint4.T preserved its binding specificity in vivo leading to tumor growth inhibition. Then, Gint4.T and CL4 aptamers were administered simultaneously intravenously in U87MG-derived mouse xenografts causing the inhibition of tumor growth with the reduction in the EGFR amount and PDGFR phosphorylation more than the single independent treatments [32]. The antitumor activity of these aptamers in vivo was also confirmed by immunohistochemical staining for the pan-proliferative marker Ki-67, revealing a significant decrease of proliferating cells in Gint4.T-treated mice, which was strongly enhanced by the combined treatment (Gint4.T + CL4) [32].

Gint4.T aptamer was also used as a chimera conjugated with an siRNA against the signal transducer and activator of transcription-3 (STAT3), leading to the formation of the Gint4.T–STAT3 aptamer–siRNA chimera named AsiC [44].

STAT3 is a signal transductor responding to EGFR and IL-6 receptor activation [52]. After its phosphorylation, STAT3 translocates into the nucleus and regulates the expression of genes involved in cell cycle, survival, hypoxia, angiogenesis, invasion, and immune response [52]. This transcription factor is often deregulated in cancers, including GBM, representing an excellent potential therapeutic target [52]. Esposito and colleagues developed the AsiC chimera (Gint4.T–siRNA by a stick-based approach), demonstrating that its binding ability to the cell target (mediated by Gint4.T) and the silencing potential (mediated by the siRNA) are preserved [53,54,55,56]. STAT3 acts as a key oncogenic factor regulating survival, proliferation, migration, and invasion of cancer cells [44,57]; accordingly, AsiC-treated cells showed a strong reduction in cell viability, the activation of programmed cell death, and a less migratory ability compared to negative controls [44].

The AsiC aptamer chimera was also tested on GBM growth in vivo. Specifically, U87MG cell-bearing mice were treated with AsiC or Gint4.T independently to compare their ability in vivo by intraperitoneal administration. The authors demonstrated an increased reduction in tumor growth in AsiC-treated mice compared to the Gint4.T treatment as tested by tumor volume and histopathological and immunohistochemical analyses [44]. AsiC-treated xenografts showed a strongly reduced cellular density and the Ki-67 positivity decreased to approximately 25%. The level of STAT3 mRNA and target genes, such as cMYC, Bcl-2, and Bcl-XL, was reduced in the tumors of treated mice as well as the level of pro-caspase 3, PARP, and Bcl-XL proteins and of programmed cell death ligand 1 (PDL1) [42].

#### 2.1.2. AS1411 Aptamer

The AS1411 aptamer is the first oligodeoxynucleotide aptamer to enter phases I and II of several cancer clinical trials [57,58,59]. It has been demonstrated that AS1411 interferes with nucleolin (NCL), whose expression is correlated with cell proliferative level and shows overexpression in GBM [60,61,62].

Specifically, Cheng and colleagues [43] demonstrated that the AS1411 aptamer by binding NCL decreased GBM cell proliferation, with p53 and cyclin A1 upregulation and Bcl-2, Akt1, and cyclin B1 downregulation. Additionally, this aptamer is also able to inhibit GBM cell migration and invasion by the downregulation of Akt1 [43]. The strong anticancer effects of AS1411 have been confirmed in vivo, in a a xenograft model of human glioma established in severe combined immunodeficient (SCID) mice. In particular, AS1411 or aptamer control sequence was subcutaneously injected in a single dose near the tumor area, every 5 days for 20 days. In vivo experiments showed a strong reduction in the tumor volume and an increased survival time of the glioma-bearing mice [43].

### 2.2. Aptamers Used as Drug Vehicles

The current clinical treatment of glioma is represented by surgery followed by radiotherapy and chemotherapy. The latter is the most commonly used method for glioma treatment [63,64,65]. The main obstacles in the treatment of gliomas are represented by the BBB and poor targeting that severely compromise the delivery of drugs to the tumor site during chemotherapy and lead to dangerous off-target effects. In the last few years, nanoparticle (NP)-based drug delivery systems demonstrated their ability to enhance cancer chemotherapy [66,67]. Although these delivery systems are very promising, they still have some limits such as poor cell targeting, premature release of the drug, and lack of real-time monitoring.

The challenge is represented by the design of nanoparticles able to cross the blood–brain/blood–tumor barrier that, thanks to the aptamers’ targeting ability, are able to exclusively reach glioma cells and release their cargo over an extended period of time to achieve an efficient therapeutic response [68,69] (Table 1).

#### 2.2.1. AS1411 Aptamer

The AS1411 aptamer, mentioned above, was largely used to decorate different kinds of NPs loaded with chemotherapeutic drug and to specifically deliver them to GBM cells [37].

Paclitaxel (PTX) is a chemotherapeutic agent largely used against various types of solid tumors including gliomas [70,71,72,73,74,75]. Unfortunately, its clinical efficacy is limited by its poor aqueous solubility, non-tumor-specific cell killing, and serious adverse side effects caused by its solvent [76]. Guo and co-authors [36] demonstrated the in vivo efficacy of the AS1411 aptamer when coupled with PTX-loaded NPs. The authors [36] used NPs derived from poly(D,L-lactic-co-glycolic acid) (PLGA). The PLGA NPs were then functionalized with polyethylene glycol (PEG), because pegylated polymeric NPs showed a significant reduction in systemic clearance compared with similar particles without PEG [77,78]. PTX-loaded PEG–PLGA NPs (PTX-NPs) were prepared and AS1411 was conjugated to the PTX-NP surface forming the Ap–PTX–NP complex exploiting aptamer AS1411–nucleolin interaction as a strategy to make PTX delivery to gliomas more specific and effective [36]. A xenograft nude mouse model with C6 glioma implanted in the armpit and an intracranial tumor model in Wistar rats were used for determining in vivo drug distribution and tumor growth delay. After intravenous administration, set according to those usually used in antiglioma therapy [72,75,79], they found a faster and stronger reduction in tumor growth compared to controls. AS1411–nucleolin-mediated recognition and internalization significantly enabled the cellular association of Ap–PTX–NP in C6 glioma cells, allowing long-term and precise in vivo tumor targeting and improving the antiglioma efficacy of PTX on both mice and rats bearing C6 glioma xenograft [36].

Luo and co-authors [40] exploited the dual targeting potential of the AS1411 aptamer, conjugating it also to a different kind of NP made of poly(L-γ-glutamyl-glutamine) (PGG). These novel PGG–PTX NPs highly improved the PTX aqueous solubility, prolonged the plasma half-life, and showed a lowered toxicity compared with PGA–PTX in mice [80,81,82], but it lacks a targeting specificity. For this reason, PGG–PTX NPs were conjugated to AS1411 that indeed enhanced the glioma treatment [36,37,83]. Moreover, the AS1411–PGG–PTX complex showed an increased ability to penetrate deeper into GBMs (2.5-fold) than the PGG–PTX NPs without the AS1411 aptamer [40]. Accordingly, in vivo studies demonstrated that AS1411–PGG–PTX nanoconjugates displayed much more accumulation of the drug and deeper penetration into GBM tissues than the PGG–PTX NPs and increased the median survival of intracranial U87MG GBM-bearing nude mice [40].

AS1411 was also used in an aptamer and peptide dual-functioned nanoparticle system [37]. Gao and co-authors [37] conjugated poly(ethyleneglycol)–poly(ε-caprolactone) (PEG–PCL) NPs and the AS1411 aptamer with a phage-displayed TGN peptide that is a specific targeting ligand of the BBB [37]. The authors [37] created the cascade targeting strategy named AsTNP to achieve high and precise brain glioma targeting. To evaluate the anticancer effect of the AsTNP system, the authors used docetaxel (DTX) that was loaded into the PEG–PCL NPs. Indeed, DTX has been widely used in the treatment of several malignancies including brain tumors [16], being an inhibitor of microtubule depolymerization [84]. In vitro, cell uptake, and three-dimensional tumor spheroid penetration studies demonstrated that AsTNP could target endothelial and tumor cells but also penetrate the endothelial monolayers and tumor cells to reach the core of tumor spheroids [37].

BALB/c nude mice bearing C6 orthotopic glioma treated by tail vein injection were used to test the in vivo potential of AsTNP to deliver DTX to tumor cells. The authors [37] demonstrated that TGN could facilitate the transportation of particles from the blood to the brain and the AS1411 aptamer could recognize glioma cells and enrich DTX-loaded particles in the tumor site. In this way, AsTNP decreased the toxicity caused by the incorrect distribution of DTX and increased the median survival of glioma-bearing mice achieving the antiglioma effect using a more precise drug delivery at a relatively low dose.

The AS1411 aptamer was also involved in the development of another dual targeting system [41]. In addition to the aptamer target (nucleolin), it has also been shown that the transferrin receptor (TfR) is overexpressed on the surface of brain capillary endothelial cells (a major component of BBB) and GBM cells [41]. Thus, several studies demonstrated that transferrin (Tf)-conjugated NPs can cross the BBB and target brain glial cells [85,86]. Based on these evidences, the combination of Tf and AS1411 aptamer nanoparticles has been proposed for the targeting of TfR- and nucleolin-expressing gliomas [41]. As therapeutic strategy, Zhu and co-authors [41] combined AS1411 and TfR with photodynamic therapy (PDT), based on the activity of a photosensitizer, which generates reactive oxygen species (ROS) under laser irradiation with a specific wavelength, leading tumor cells to apoptosis. Moreover, it is well known that targeted chemotherapy combined with PDT can significantly improve cancer treatment [87]. The authors [41] selected the anticancer agent RBT [Ru(bpy)_2_(tip)]^2+^, which is a high-efficiency photosensitizer for the photodynamic tumor therapy [88]. To refine the system, the authors used mesoporous ruthenium nanoparticles. Indeed, among the different inorganic metal nanoparticles used to treat and diagnose brain gliomas [89,90,91,92,93], the ruthenium nanoparticles are particularly appealing due to their good biocompatibility [41]. The authors [41] optimized the ruthenium nanoparticles obtaining mesoporous ruthenium nanoparticles (MRN) increasing the loading ability of antitumor drugs (28.2%) [94]. In addition, since glutathione (GSH) levels in tumor cells are much higher than in normal cells, drug release based on endogenous GSH is considered to be the most efficient strategy and disulfide bonds are the most commonly used part of the GSH trigger system [95,96,97,98]. Standing on these evidences, the authors [41] proposed the use of MRN RBT-loaded NPs covalently bound to both Tf and AS1411 with the addition of the disulfide bonds to obtain a dual-targeted nanomedicine delivery system for drug delivery to gliomas, which they called RBT@MRN-SSTf/Apt [41]. In vitro, the RBT@MRN-SSTf/Apt complex penetrates deeply and has a significant inhibitory effect on 3D tumor cell spheres. To assess the in vivo therapeutic potential, U87MG orthotopic tumor-bearing nude mice were treated with RBT@MRN-SSTf/Apt combined with laser [41]. In vivo data demonstrated that the median survival of tumor-bearing nude mice was significantly prolonged with minimal weight loss [41]. This and other studies provide new possibilities for the design of dual-targeted NP-mediated drug delivery systems combined with photodynamic therapy.

#### 2.2.2. GMT8 Aptamer

Among the aptamers developed through whole-cell SELEX, the GMT8 aptamer has been found to be the one with the highest binding affinity for GBM cells even though its target is still unknown [8]. Therefore, it has been used as an efficient ligand for GBM-targeting therapy to improve drug delivery to GBM cells and enhance tumor penetration [38].

The GMT8 aptamer was also enhanced employing the non-cytotoxic polyethylene glycol–poly ε-caprolactone (PEG–PCL) NPs. Specifically, GMT8 was conjugated on the surface of PEG–PCL NPs loaded with DTX forming the delivery system named ApNP [38]. GBM cells treated with only DTX, DTX-loaded NPs, or ApNP were analyzed and the nuclear fragmentation (suggestive of apoptosis) was found higher in cells treated with ApNP compared to those treated with only DTX or with DTX-loaded NPs [38]. The in vivo therapeutic potential of ApNP was tested in orthotopic U87MG GBM-bearing nude mice. The animals treated with fluorescent NPs and ApNP showed higher brain fluoresce in ApNP-treated mice, with the GMT8 aptamer increasing mice survival time compared to unconjugated NPs [38].

#### 2.2.3. ATP Aptamer

An and co-authors [39] developed a targeting delivery system based on an ATP aptamer coupled with a modified substrate of the amino acid transporter LAT1 and a GSH responsive molecule [39] acting as dual-release regulating factors for the efficient doxorubicin (DOX) delivery into GBM cells. The ATP DNA aptamer is a 25-base single-stranded oligodeoxynucleotide selected from a random-sequence DNA pool and showing high affinity for ATP [39]. To regulate DOX delivery, the ATP aptamer was hybridized with its cDNA, forming a DNA scaffold as a DOX carrier. DOX can specifically intercalate into the GC pairs of the DNA scaffold, yielding DOX/ATP aptamer complex without changing the duplex structure of the DNA scaffold and allowing DOX release depending on ATP concentration. Indeed, in vitro, only the ATP amount of tumor cells was able to trigger the DOX release, while it was not induced by the low extracellular concentration, ensuring DOX release only to the tumor cells [39].

The system was then improved using another targeting molecule named 3CDIT, a substrate derivate of LAT1 that is an amino acid transporter expressed by both the BBB and glioma cells [39]. Then, considering the great disparity in GSH levels between extracellular and intracellular compartments, the 3CDIT substrate was decorated onto a GSH-responsive polymer (pOEI), yielding a 3CDIT-targeting pOEI complex that was afterwards condensed with the DOX/ATP complex forming the 3CDIT-pOEI/DOX/ATP aptamer delivery system [39].

To test its therapeutic efficacy, the 3CDIT-pOEI/DOX/ATP complex was injected through tail vein in a glioma nude mice model with stable luciferase expression. In vivo, this new delivery system demonstrated an outstanding glioma accumulation, DOX release, and antitumor therapeutic effect, without systemic toxicity, thereby opening a new scenario for safe and efficient glioma chemotherapy [39].

### 2.3. Aptamers Able to Enhance GBM Therapy Efficacy

As reported above, GBM is one of the most common and deadliest brain tumors. Surgical resection followed by radiotherapy (postoperative fractionated external-beam radiotherapy started within 6 weeks of surgery with 60 Gy) [99] and chemotherapy (using temozolomide (TMZ)) is the standard therapy for this type of cancer. The effects of this traditional therapeutic strategy are very limited due to the abnormal migration and invasion ability of GBM cells as well as their resistance to chemo- and radiotherapy. Therefore, the discovery of a drug able to inhibit GBM cell proliferation, migration, and invasion and decrease chemo- and radioresistance is a crucial step for developing an efficient GBM treatment [100,101]. To date, a few aptamers have been able to increase the efficacy of GBM existing therapies in vivo (Table 1).

#### 2.3.1. Aptamers Enhancing Radiotherapy Sensitivity

##### U2 Aptamer

The first is the DNA aptamer, named U2, obtained by cell-SELEX technology using GBM cells overexpressing epidermal growth factor receptor variant III (EGFRvIII) [102]. EGFRvIII is the most common gain-of-function mutation observed in 50% of GBM patients. This mutation has reduced constitutive EGFR-mediated signals compared to the wild-type EGFR [32,103,104]. In addition, EGFRvIII expression has been correlated to chemo- and radioresistance in GBM patients [19,105,106]. By flow cytometry and immunofluorescence methods, U2 aptamer has been demonstrated to bind specifically EGFRvIII-expressing GBM cells and the aptamer–receptor complex was internalized into the cells through the endosome recycling pathway [102]. Since the EGFRvIII suppression led to a reduction in cancer cell proliferation, migration, and invasion [102,107], Zhang and co-authors [102] analyzed the U2 effects on these typical cancer features. They demonstrated that the aptamer caused a time- and dose-dependent increase in apoptosis rate and reduced the migration capability of cancerous cells diminishing the phosphorylation level of EGFRvIII.

Most importantly, the U2 aptamer enhances the radiosensitivity of EGFRvIII-expressing cells by decreasing the phosphorylation of DNA repair effectors and enhancing the DNA repair process [102]. In a nude mouse model bearing EGFRvIII-expressing GBM cells, U2 aptamer dramatically reduced tumor volume and showed a significant antitumor effect compared to the control [102].

##### NOX-A12 Aptamer

Radiotherapy is an important component of the GBM treatment able to abrogate local angiogenesis inducing the tumor mass to activate the neovasculogenesis pathway, which involves de novo growth of blood vessels [108]. Starting from this evidence, Brown and colleagues [109] speculated that tumor recurrence could be markedly reduced by inhibition of the circulating pro-angiogenic CD11b+ myelomonocytes that express high levels of stromal cell-derived factor 1 (SDF1) playing a key role in angiogenesis by recruiting endothelial progenitor cells through a CXCR4/CXCR7 dependent mechanism.

Therefore, the most effective strategy for preventing post-irradiation vasculogenesis in GBM would be to block SDF-1 receptors (CXCR4 and CXCR7).

A PEGylated-L-oligoribonucleotide aptamer (the so-called Spiegelmer) NOX-A12 (olaptesed pegol) that binds with high affinity SDF-1 was used to achieve this purpose [109]. NOX-A12 consists of 45 L-enantiomeric RNA nucleotides and carries a 40-kDa polyethyleneglycol (PEG) modification at its 5′-end to increase plasma permanence time. The non-natural L-nucleotides confer biostability to the molecule because L-oligonucleotides are not recognized by nucleases. Additionally, their mirror-image nature renders Spiegelmers immunologically passive, with a low risk of neutralizing antibodies and no Toll-like receptor activation.

To test NOX-A12 in vivo, the authors used N-ethyl-N-nitrosourea (ENU)-induced brain tumors in the Sprague-Dawley rat, a model that has been proven to be extremely resistant to anticancer therapy [110]. The authors demonstrated that NOX-A12-mediated SDF-1 blockade was indeed effective in inhibiting or delaying GBM recurrences following irradiation in this model.

Considering that NOX-A12 was delivered in vivo following brain irradiation at doses and time periods that can be safe and well tolerated in humans, this aptamer was engaged for a clinical trial in 2019. The purpose of the ongoing study (ClinicalTrials.gov identifier NCT04121455) is to obtain exploratory information on the safety and efficacy of NOX-A12, in combination with radiation therapy in patients with newly diagnosed GBM either not amenable to resection (biopsy only) or after incomplete tumor resection.

##### AS1411 Aptamer

With the nanotechnology improvements, different metal nanomaterials have been also developed to enhance the antitumor efficacy of radiotherapy. In particular, silver nanoparticles (AgNPs) showed excellent radiosensitizing properties that have been confirmed on glioma cells in vitro and in vivo [46,111,112,113,114]. Thus, in order to enhance the radiosensitivity of tumor cells, Zhao and co-authors [46] conjugated NPs with polyethylene glycol (PEG) and the AS1411 aptamer (AsNPs), and demonstrated that conjugated AsNPs led to a lower cytotoxicity and enhanced the AgNP endocytosis into C6 GBM cells. In addition, only the PEG–AS1411 conjugated nanoparticles reached the spheroid core, suggesting an improved penetration ability. Since previous studies indicated the apoptosis induction as a potential mechanism of radiosensitization, the authors demonstrated that AsNPs carried out their radiosensitizing function triggering the apoptotic response [46]. For the in vivo radiosensitization experiments, AsNPs were conjugated with the Cy5 fluorophore and systematically administrated to nude mice bearing intracranial glioma. Cy5–AsNPs were able to accumulate into the tumor and, most importantly, the median survival time of mice, treated with AsNPs plus irradiations, was significantly elongated. Notably, systemic toxicity was not observed in injected mice [46].

#### 2.3.2. Immune-Modular Aptamers

Another class of aptamers enhancing GBM therapy efficacy is represented by aptamers able to modulate the immune response. Immunotherapy is based on the use of monoclonal antibodies, immune adjuvants, and vaccines against oncogenic viruses [115]. Immune-modular aptamers showed a high targeted delivery capacity, conferring on them less off-target side effects and a good plasticity. For these reasons, they are considered excellent potential therapeutic tools [116]. Usually, in order to reduce toxicity, this class of aptamers is targeted to the tumor cells by the conjugation to a second aptamer, which is able to precisely bind to cancer surface receptors, pledging an activity only against cancer cells [117] (Table 1).

##### VEGF-4-1BB Bi-specific Aptamer

Among the immune-modular aptamers, 4-1BB was tested in an oncogene-induced GBM model in vivo. 4-1BB was a co-stimulatory receptor, stimulating activated CD8+ T-cell survival, expansion, and differentiation into memory cells [118], thereby enhancing tumor immunity and inhibiting cancer growth [119,120,121]. Different studies reported that systemic treatments with anti-4-1BB antibody (Ab), synergized with vaccination and other immunotherapies, showed tumor growth inhibition in mice [116,120,122,123,124,125,126]. Unfortunately, anti-4-1BB Ab causes immune anomalies due to polyclonal activation of CD8+ T cells and consequent overproduction of IFNγ and TNF [45,122,124,127]. To bypass this block, Schrand and colleagues [45] tested the efficacy of a bi-specific aptamer-based approach. A specific targeting based on an aptamer developed against the broadly expressed stromal product, the vascular endothelial growth factor (VEGF) [122], was used to bind GBM stroma, while the 4-1BB aptamer was used to bind the T-cell co-stimulatory receptor in order to induce the immune response activation. The in vivo efficacy of the VEGF–4-1BB system has been tested in high-grade glioma murine models overexpressing PDGFRβ and STAT3 in cancer precursor cells of newborn mice. The treatment with this bi-specific aptamer (VEGF–4-1BB) led to an enhanced mice survival, confirming the conjugate antitumoral efficacy and suggesting its ability to cross the BBB [45].

## 3. Discussion

Aptamers show many features that make them promising agents for cancer/GBM treatment. These short single-stranded DNA or RNA oligonucleotides are able to bind to target molecules with high affinity in three-dimensional shapes like antibodies or peptides but with additional benefits.

Monoclonal antibodies (mAbs) are immunogenic peptides/proteins that can bind to specific epitopes inducing biochemical reactions. Multiple studies have reported the importance of mAbs in cancer; for example, an anti-mouse transferrin antibody fused with the GDNF (cTfRmAb-GDNF) can be delivered to the brain in vivo [128]. Although there are few examples of mAbs in clinical trials, the presence of antibodies in the brain has also been linked to neurological disorders such as psychosis [129].

Considering the above, aptamers can be an important alternative. Indeed, aptamers possess several advantages compared to antibodies. For example, aptamers do not produce an immune response, can be selected for a wider range of targets including highly toxic compounds, are generated in a relatively short time compared to antibodies, and are characterized by a high cell and tissue permeability due to their small size (~12–30 kDa) compared to the typical IgG antibody (~150–170 kDa). Moreover, aptamers are chemically synthesized, and there is no risk of biological contamination and batch-to-batch consistency and, finally, they do not involve animals.

Regarding peptides, which are short linear chains of amino acids (aa) usually <50 aa in length, they can bind, modulate, and inhibit specific proteins of interest; for example, they can inhibit a specific interaction between two proteins. In cancer treatment, these peptides can be used in a variety of ways, including carrying cytotoxic drugs, hormones, radionuclides, and vaccines similarly to aptamers [130]. Peptides, similarly to aptamers, have several important advantages over antibodies, such as they have a small size and are relatively easier to synthesize than recombinant antibodies. However, they have several disadvantages compared to aptamers, such as low bioavailability, metabolic liability, poor cell permeability, immunogenicity, high degradability in vivo by proteases, strong delivery problems, and less versatility compared to aptamers [131].

Considering the above, aptamers can be considered as highly promising molecules for future clinical application; however, despite several advantageous features, some limitations must be considered. The most important one is their susceptibility to digestion by nucleases, especially in vivo. At present, several technical approaches have been utilized to improve aptamer nuclease resistance, the most promising being the substitution of the 2′OH of the sugar backbone of RNAs with fluoro, amino, or methoxy functional groups or the use of locked nucleic acids (LNAs) [132].

During the last few years, a large number of aptamers have been generated against GBM cell membrane proteins; some of them are able to cross the BBB and have been proven to be efficient as therapeutic agents in GBM in vivo models. Among them, we note the following: (i) aptamers developed as anti-GBM tools blocking cancer progression; (ii) aptamers used as drug vehicles for antitumoral molecules or for nanoparticles and their derivatives, representing novel drug delivery systems; (iii) aptamers used as adjuvants to increase the efficacy of GBM existing therapies in vivo; and (iv) aptamers able to modulate the immune response, enhancing tumor immunity and inhibiting cancer growth.

However, despite the availability of several aptamers for GBM therapy, only the NOX-A12 aptamer is, at present, recruited for a clinical trial started in 2019.

## 4. Conclusions

The exponential increase in aptamer research and their growing applications in multiple fields of biological and clinical research clearly indicate them as strong rivals of antibodies and peptides as molecules with specific binding characteristics and therapeutic potential. In this scenario, future studies on aptamer-based therapy are necessary to open novel possibilities in the advanced clinical therapy of GBM.

## Figures and Tables

**Table 1:** The characteristics of aptamers used to treat GBM in vivo.

Aptamer Name	Conjugate Name	Aptamer Target	Producing Method	Oligonucleotides	Modifications	References
Anti-tumoral activity Gint4.T CL4 AS1411	Unconjugated	PDGFRβ EGFR Nucleolin	Cell-SELEX Cell-SELEX DNA oligonucleotides screening	RNA aptamer RNA aptamer DNA aptamer	Unmodified	[42] [42] [43]
Anti-tumoral molecules vehicles Gint4.T	AsiC	PDGFRβ	Cell-SELEX	RNA aptamer	STAT3 siRNA	[44]
Drugs vehicles AS1411 AS1411 AS1411 AS1411 GMT8 ATP aptamer	Ap-PTX-NP AS1411-PGG-PTX AsTNP RBT@MRN-SSTf/Apt ApNp 3CDIT/pOEI/DOX/ATP aptamer	Nucleolin A-172 cell line (target unknown) Intracellular ATP	DNA oligonucleotides screening Cell-SELEX Conventional SELEX	DNA aptamer DNA aptamer DNA aptamer	PEG-PLGA NPs loaded with PTX PEG-PGG Nps loaded with PTX TGN + PEG-PCL Nps loaded with DTX Tf + SS + MRN NPs loaded with RBT PEG-PCL Nps loaded with DTX 3CDIT + pOEI + DOX	[36] [40] [37] [41] [38] [39]
Enhancing therapy efficacy U2 NOX-A12 (Olaptesed pegol) AS1411 VEGF aptamer + 4-1BB	^188^Re-U2 NOX-A12 (Olaptesed pegol) Cy5-AsNPs VEGF-4-1BB	EGFRvIII SDF-1 Nucleolin VEGF + CD8^+^ cells	Cell-SELEX Conventional SELEX DNA oligonucleotides screening Conventional SELEX	DNA aptamer RNA aptamer DNA aptamer RNA aptamer	^188^Re PEG Cy5-AgNPS-PEG Unmodified	[102] [109] [46] [45]
